# Evaluating the long-term impact of COVID-19-associated public health interventions on zoonotic and vector-borne diseases in China: an interrupted time series analysis

**DOI:** 10.1186/s12967-024-04855-y

**Published:** 2024-01-20

**Authors:** Yongbin Wang, Siyu Qing, Xianxiang Lan, Lun Li, Peiping Zhou, Yue Xi, Ziyue Liang, Chenguang Zhang, Chunjie Xu

**Affiliations:** 1 Department of Epidemiology and Health Statistics, School of Public Health, School of Medical Technology, The First Affiliated Hospital, Xinxiang Medical University, No. 601 Jinsui Road, Hongqi District, Xinxiang, 453003 Henan People’s Republic of China; 2 Beijing Key Laboratory of Antimicrobial Agents/Laboratory of Pharmacology, Institute of Medicinal Biotechnology, Chinese Academy of Medical Sciences & Peking Union Medical College, Beijing, 100050 China

**Keywords:** Zoonotic and vector-borne diseases, COVID-19, Dynamic zero-case policy, ARFIMA, Intervention, Interrupted time series analyses

## Abstract

**Background:**

The long-term impact of COVID-19-associated public health interventions on zoonotic and vector-borne infectious diseases (ZVBs) remains uncertain. This study sought to examine the changes in ZVBs in China during the COVID-19 pandemic and predict their future trends.

**Methods:**

Monthly incidents of seven ZVBs (Hemorrhagic fever with renal syndrome [HFRS], Rabies, Dengue fever [DF], Human brucellosis [HB], Leptospirosis, Malaria, and Schistosomiasis) were gathered from January 2004 to July 2023. An autoregressive fractionally integrated moving average (ARFIMA) by incorporating the COVID-19-associated public health intervention variables was developed to evaluate the long-term effectiveness of interventions and forecast ZVBs epidemics from August 2023 to December 2025.

**Results:**

Over the study period, there were 1,599,647 ZVBs incidents. HFRS and rabies exhibited declining trends, HB showed an upward trajectory, while the others remained relatively stable. The ARFIMA, incorporating a pulse pattern, estimated the average monthly number of changes of − 83 (95% confidence interval [CI] − 353–189) cases, − 3 (95% CI − 33–29) cases, − 468 (95% CI − 1531–597) cases, 2191 (95% CI 1056–3326) cases, 7 (95% CI − 24–38) cases, − 84 (95% CI – 222–55) cases, and − 214 (95% CI − 1036–608) cases for HFRS, rabies, DF, HB, leptospirosis, malaria, and schistosomiasis, respectively, although these changes were not statistically significant besides HB. ARFIMA predicted a decrease in HB cases between August 2023 and December 2025, while indicating a relative plateau for the others.

**Conclusions:**

China's dynamic zero COVID-19 strategy may have exerted a lasting influence on HFRS, rabies, DF, malaria, and schistosomiasis, beyond immediate consequences, but not affect HB and leptospirosis. ARFIMA emerges as a potent tool for intervention analysis, providing valuable insights into the sustained effectiveness of interventions. Consequently, the application of ARFIMA contributes to informed decision-making, the design of effective interventions, and advancements across various fields.

**Supplementary Information:**

The online version contains supplementary material available at 10.1186/s12967-024-04855-y.

## Background

Coronavirus disease 2019 (COVID-19), caused by the SARS-CoV-2 virus, is a highly contagious respiratory illness [1]. It was initially identified in Wuhan, China, in December 2019 [1]. Since then, the disease has rapidly spread worldwide, resulting in a devastating global pandemic [2]. As of 11 November 2023, there have been a total of 771,820,937 confirmed cases and 6,978,175 deaths reported in over 200 countries, areas, or territories [2]. Although the WHO declared COVID-19 no longer a global health emergency on 5 May 2023, this pandemic has brought about immense tragedy and had a profound impact on global health and society [2]. During the COVID-19 pandemic, China implemented a dynamic “zero-COVID” policy aimed at achieving zero local transmission of the virus within the country [3]. This policy involved strict measures such as widespread testing, contact tracing, quarantine, mask-wearing, social distancing, travel restrictions, and localized lockdowns [3].

Studies have demonstrated the effectiveness of COVID-19-related interventions in preventing the potential spread of the virus and ensuring public safety [4]. However, these stringent measures have also had an impact on the surveillance and transmission of other infectious diseases. Recent research has indicated that these COVID-19-associated interventions have led to a decrease in the morbidity of many infectious diseases such as tuberculosis, influenza, mumps, sexually transmitted diseases, dengue fever (DF), etc. [5–10]. However, the above-mentioned studies only investigated the short-run effect of COVID-19-associated interventions on diseases (i.e., the year 2020) [11], and most focused on respiratory diseases and sexually transmitted diseases in local regions [8, 9]. The ongoing COVID-19-associated interventions have persisted until 2023 but have varied in intensity in different countries as the pandemic situation fluctuated. Zoonotic and vector-borne infectious diseases (ZVBs) are a global public health concern [6, 11, 12], including in China. These diseases are primarily transmitted from animals to humans, either through direct contact, consumption of contaminated animal products, or exposure to vectors carrying the pathogens [12]. They can have significant impacts on human health, leading to morbidity, mortality, and economic burdens [12]. However, there is currently no comprehensive study examining the long-term impact of public health interventions against COVID-19 on ZVBs. Therefore, this study aimed to investigate the long-term impact of COVID-19-associated interventions on ZVBs and forecast the epidemics until December 2025 using a new interrupted time series (ITS) method of autoregressive fractionally integrated moving average (ARFIMA). The availability of such evidence would be invaluable in guiding public health policies and selecting methods for the prevention and control of diseases in the future.

## Materials and methods

### Data extraction

The monthly incidence cases of seven ZVBs (Hemorrhagic fever with renal syndrome [HFRS], Rabies, DF, Human brucellosis [HB], Leptospirosis, Malaria, and Schistosomiasis) from January 2004 to July 2023 were obtained from the Chinese Center for Disease Control and Prevention (CDC) and the population data were taken from the Statistical Yearbook 2022. The diagnoses of ZVBs incidents were based on the respective guidance criteria issued by the National Health Commission of China (http://www.nhc.gov.cn/wjw/s9491/wsbz.shtml).

### Establishment of the ARFIMA

ITS analysis is a useful tool to evaluate the impact of interventions by comparing trends before and after the interventions [13]. Two commonly used models for this analysis are autoregressive integrated moving average (ARIMA) and segmented regression models (SRM) that assume a simple linear trend in series [13, 14]. However, epidemics of infectious diseases often have secular trends, seasonality, and random variations, and thus the direct application of SRM compromises the accuracy of assessing intervention effects [14]. In such cases, using ARIMA would be more suitable for a more accurate analysis [14]. This model is typically represented as ARIMA (p, d, q) (P, D, Q) s, where p denotes the number of autoregressive (AR) terms; d represents the degree of differencing; q signifies the number of moving average (MA) terms; P, D, and Q correspond to the seasonal terms (SAR, SMA, and seasonal differencing); and s is the number of periods in a season. Although the ARIMA has successfully been applied in ITS analysis thanks to its comprehensive consideration of the trend, seasonal, and random components in a series [10, 14–18], it is designed for modelling short-term fluctuations and the use of integer integration in ARIMA can lead to over-differencing and the loss of useful features, potentially harming predictive ability [19]. Studies have shown that epidemics of infectious diseases often exhibit long memory properties [20]. Intriguingly, ARFIMA extends the ARIMA by including a fractional integration (d_*f*_), which allows to capture both short and long memory simultaneously [20, 21]. Different ranges of d_*f*_ indicate various characteristics of a series. Typically, d_*f*_ ∈ (− 1, 0.5) is used, where d_*f*_ ∈ (− 0.5, 0) suggests series invertibility, d_*f*_ ∈ (− 1, − 0.5) indicates anti-persistence, d_*f*_ = 0 represents short memory and mean-reverting process, and d_*f*_ ∈ (0, 0.5) signifies long-range persistence [20, 21]. The usual ARFIMA consists of six components: nonseasonal AR, fractional differencing (d_*f*_), MA, and seasonal versions of these three components (SAR, D_*f*_, and SMA) [20]. This model is denoted as ARFIMA (p, d^*^, q) (P, D^*^, Q) _S_, where d^∗^  = d + d_*f*_ or D^∗^  = D + D_*f*_, d_*f*_ or D_*f*_ represents fractional integration, and d represents the integer part (d ≥ 0) [20].

The Hurst exponent (H) is a statistical measure used to analyze the long-term memory and predictability of a time series, quantifying the degree of persistence or anti-persistence [22]. The relationship between H and d_*f*_ is denoted as d_*f*_ or D_*f*_ = H—0.5, where H > 0.5 indicates a persistent series, H < 0.5 suggests an anti-persistent series, and H = 0.5 represents a random walk [20]. The computation of H involves techniques such as rescaled range (R/S) analysis, correct R/S, or detrended fluctuation analysis [23]. In this study, the corrected R/S method was used to determine if different ZVBs incidences display long-range properties [23]. Constructing the ARFIMA model involves selecting the best parameters by maximizing log-likelihood (LL) and minimizing the Akaike Information Criterion (AIC) and Bayesian Information Criterion (BIC) [21]. Estimating the parameters and conducting model diagnoses followed the four procedures [20, 21]. First, the stationarity of the ZVBs incidence series was assessed using a Kwiatkowski-Phillips-Schmidt-Shin (KPSS) unit root test [24]. If *p* < 0.05, indicating that the series would not be stationary due to the presence of a unit root, then differencing was required to achieve stationarity; otherwise, it was not necessary. Second, the appropriate ARFIMA structure was identified by examining the autocorrelation function (ACF) and partial autocorrelation function (PACF) plots, which provided rough estimates for the values of p, q, P, and Q [20]. Various combinations of these values were considered, and the best model was selected based on maximizing LL and minimizing the AIC, corrected AIC (CAIC), and BIC [20]. Third, model checks were performed to evaluate whether the resulting residuals behaved like a white noise series, using analyses such as the Ljung-Box Q test, autocorrelogram, and partial autocorrelogram [20]. Finally, once the best model passed the diagnostic tests, it could be used to forecast.

### Impact patterns of interventions using ARFIMA

The objective of ITS analysis when evaluating interventions is to estimate the effect of implementing the interventions on a specific outcome [14]. Often, a comparison can be conducted between the pre-intervention and post-intervention periods to determine whether there was a significant difference in the post-intervention phase compared to the pre-intervention phase. Three main types of impacts can be observed: step change, pulse, and ramp [14]: A step change, or level shift, refers to a sudden and sustained change where the series is immediately shifted either upward or downward by a specific value following the intervention; A pulse refers to a sudden and transient change that becomes apparent for one or more time points immediately after the intervention and subsequently reverts to the baseline level; A ramp effect represents a change in slope that promptly materializes after the intervention.

The preferable shape of the intervention impact should ideally be hypothesized in advance. This choice hinges on multiple factors, encompassing the nature of the intervention (be it temporary or ongoing), and the specific outcome under estimation [14]. In contrast, interventions that are continuous or permanent are more prone to exert long-term effects, which can manifest as either immediate or gradual shifts. In some scenarios, the most accurate representation of the intervention's impact might involve a combination of impact variables [14]. For instance, it is common to find both a step change and a change in slope coexisting within the same analysis. When faced with multiple potential models, the AIC and BIC can help select the most appropriate combination of impact variables [14].

In this study, the overall timeframe for the COVID-19-associated interventions ranged from January 2020 to January 2023, considering the impact patterns of COVID-19-associated interventions that work immediately during the interventions and decline gradually in the post-interventions, supposing that there would be a pulse change following an immediate rise during the COVID-19 outbreak (coding as “1”) and then decay by a fixed value (such as “0.8, 0.6, 0.4…”) after the COVID-19 outbreak, while at all other times, it remains at “0” [13]. To test the sufficiency and appropriateness of such a hypothesis, we also compared it with other intervention types (e.g., ramp and step) or the combinations of different intervention types (e.g., pulse + ramp, step + ramp, step + pulse) for HFRS. A greater value of LL and a lower value of AIC, CAIC, BIC, and mean absolute percentage error (MAPE) corresponded to the preferred change patterns across all intervention types.

### Statistical analysis

The Hodrick-Prescott (HP) method was used to decompose the trend and cyclicity of the ZVBs incidence series [25], and the seasonal index (SI) was calculated using classical multiplicative decomposition to suggest the extent to which the morbidity for a specific phase is typically higher or lower than the mean [26]. The ARFIMA was built using the “arfima” packages in R (version 4.2.0, R Development CoreTeam, Vienna, Austria). Statistical significance was set at *p* < 0.05.

## Results

### Descriptive analysis

**Fig. 1:**
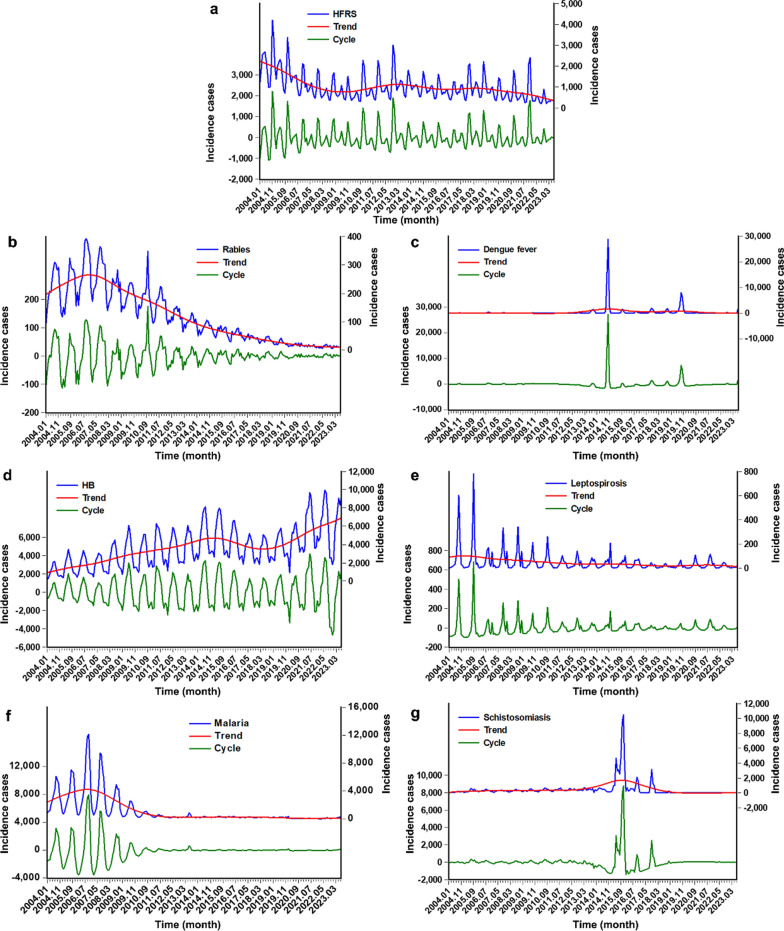
The original series and the trend (the y-axis on the right) and cycle (the y-axis on the left) components decomposed by the HP method for a the HFRS incidence series, b the Rabies incidence series, c the DF incidence series, d the HB incidence series, e the Leptospirosis incidence series, f the Malaria incidence series, and g the Schistosomiasis incidence series. As depicted above, together there was a reduction in HFRS and rabies incidences; there was a relative stableness in DF, leptospirosis, malaria, and schistosomiasis incidences; there was a notable increase in HB incidence

A total of 1,599,647 (yearly incidence rate: 5.961 per 100,000 persons) ZVBs incidents were reported from January 2004 to July 2023 in China, with monthly 6807 incidents (monthly incidence rate: 0.497 per 100,000 persons). Of them, 236,854 HFRS cases, 28,028 rabies cases, 97,843 DF cases, 853,491 HB cases, 10,848 leptospirosis cases, 269,193 malaria cases, and 103,390 schistosomiasis cases. The morbidity rates were 0.905, 0.108, 0.371, 3.104, 0.042, 1.033, and 0.399 per 100,000 persons, respectively (Additional file 1: Fig. S1). The trend and cyclicity decomposed by HP are illustrated in Fig. 1, showing a clear trend of decreasing in HFRS and rabies incidences, a relative trend of stableness in DF, leptospirosis, malaria, and schistosomiasis incidences, and a clear trend of increasing in HB incidence. Also, from the data in Additional file 1: Table S1, there was a notable seasonality with a certain degree of variation in different ZVBs.

### Determining the preferred impact patterns of interventions

Table 1 shows the information criteria for the different impact patterns of COVID-19-associated public health interventions, it is apparent that the pulse change was the preferred as it produced a lower value of AIC (3037), CAIC (3037.53), BIC (3060.82), and MAPE (16.7%), alongside a greater value of LL (-1511.50) compared with those of the change patterns of step, ramp, pulse + ramp, step + ramp, and step + pulse. Accordingly, a pulse pattern was used in our analysis.

**Table 1:** Combinations of different change patterns with the corresponding information criterion

Change patterns	AIC	CAIC	BIC	LL	MAPE %
Pulse	3037.00	3037.53	3060.82	− 1511.50	16.7
Step	3037.18	3037.70	3061.00	− 1511.59	16.8
Ramp	3037.24	3037.77	3061.06	− 1511.62	16.8
Pulse + Ramp	3039.00	3039.68	3066.23	− 1511.50	16.7
Step + Ramp	3039.16	3039.84	3066.38	− 1511.58	16.8
Step + Pulse	3038.77	3039.45	3065.99	− 1511.39	16.7

### Model development and checking

The KPSS statistic of 2.38 > 0.46 (critical value) for the HFRS incidence (corrected R/S = 0.86) showed that it was non-stationary (*p* < 0.05), and then differencing seasonally (0.78 > 0.46) and non-seasonally (0.02 < 0.46) once, indicating a stationary series (*p* > 0.05). By depicting the ACF and PACF plots of this stationary series and comparing the information criteria of different modes (Additional file 1: Figure S1, Additional file 1: Tables S2–S3), we identified the ARFIMA (2,0.462,1) (1,0.379,1)_12_ as the best model because this mode generated the minimum values of AIC (2574.856) and BIC (2609.451), along with the maximum value of LL (-1277.43) across the possible modes (Additional file 1: Table S3). Further checks indicated that the estimated parameters were statistically significant (*p* < 0.05) (Table 2), and the residuals behaved like a white noise series because there were no statistical difference under the Ljung-Box Q test ($\chi^{2}$ = 0.004, *p* = 0.951) and no significant spikes in the ACF and PACF plots (Additional file 1: Fig. S3a). These diagnoses confirmed the adequacy of this selected model. Likewise, following the modelling steps of the ARFIMA, we could identify the optimal ARFIMA(1,0,1)(1,0.06,2)_12_ for rabies (corrected R/S = 0.84), ARFIMA(0,0,1)(0,0.072,0)_12_ for DF (corrected R/S = 0.6), ARFIMA(1, − 0.732, (1,2,3,4,6))(1,0.436,1)_12_ for HB (corrected R/S = 0.85), ARFIMA(0,0,2)(2,0.163,1)_12_ for leptospirosis (corrected R/S = 0.68), ARFIMA(2,− 0.713,0)(1,0.29,1)_12_ for malaria (corrected R/S = 0.97), and ARFIMA(4,0,0)(1,0,0)_12_ for schistosomiasis (corrected R/S = 0.8), and model checking results are given in Table 2, Additional file 1: Figs. S4–S6 and S3b–f.

**Table 2:** The determined optimal ARFIMA for different zoonotic and vector-borne diseases

Variables	Estimates	S.E	t	*p*	AIC	BIC	LL	Ljung-Box	
								Statistics	*p*
ARFIMA (2,0.462,1) (1,0.379,1)_12_ for HFRS incidence series between January 2004 and July 2023									
AR1	0.752	0.138	5.459	< 0.001	2574.856	2609.451	− 1277.43	0.004	0.951
AR2	− 0.397	0.076	− 5.197	< 0.001					
MA1	0.285	0.152	1.875	0.031					
SAR1	0.966	0.04	24.204	< 0.001					
SMA1	0.929	0.079	11.818	< 0.001					
Pulse change	− 82.137	138.167	− 0.594	0.552					
ARFIMA (1,0,1) (1,0.06,2)_12_ for rabies incidence series between January 2004 and July 2023									
AR1	0.941	0.024	38.618	< 0.001	1518.222	1552.818	− 749.111	0.662	0.416
MA1	0.417	0.085	4.916	< 0.001					
SAR1	0.914	0.064	14.309	< 0.001					
SMA1	0.649	0.207	3.135	0.002					
SMA2	− 0.163	0.079	− 2.075	0.038					
Pulse change	− 2.038	15.773	− 0.129	0.897					
ARFIMA (0,0,1) (0,0.072,0)_12_ for dengue fever incidence series between January 2004 and July 2023									
MA1	− 0.59	0.049	− 11.923	< 0.001	3547.62	3568.38	− 1767.81	0.705	0.401
Pulse change	− 467.039	542.679	− 0.861	0.389					
ARFIMA (1,-0.732,(1,2,3,4,6) (1,0.436,1)_12_ for HB incidence series between January 2004 and July 2023									
AR1	0.993	0.011	90.681	< 0.001	3037.48	3085.92	− 1504.74	0.009	0.926
MA1	− 0.651	0.103	− 6.301	< 0.001					
MA2	− 0.419	0.108	− 3.874	< 0.001					
MA3	− 0.399	0.086	− 4.66	< 0.001					
MA4	− 0.22	0.09	2.447	0.014					
MA6	− 0.12	0.066	− 1.825	0.035					
SAR1	− 0.828	0.347	− 2.386	0.017					
SMA1	− 0.8	0.367	− 2.183	0.029					
Pulse change	2190.13	579.051	3.783	< 0.001					
ARFIMA (0,0,2) (2,0.163,1)_12_ for Leptospirosis incidence series between January 2004 and July 2023									
MA1	− 0.398	0.068	− 5.901	< 0.001	1830.26	1864.86	905.13	0.064	0.8
MA2	− 0.143	0.068	− 2.119	0.034					
SAR1	0.688	0.135	5.10	< 0.001					
SAR2	0.224	0.11	2.034	0.022					
SMA1	0.40	0.163	2.452	0.014					
Pulse change	6.882	15.675	0.439	0.661					
ARFIMA (2,− 0.713,0) (1,0.29,1)_12_ for malaria incidence series between January 2004 and July 2023									
MA1	1.654	0.188	8.785	< 0.001	1430.06	1457.91	− 706.031	0.001	0.971
MA2	− 0.659	0.183	− 3.605	< 0.001					
SAR1	− 0.996	0.01	− 98.859	< 0.001					
SMA1	− 0.985	0.024	− 40.556	< 0.001					
Pulse change	− 83.131	70.378	− 1.181	0.238					
ARFIMA (4,0,0) (1,0,0)_12_ for Schistosomiasis incidence series between January 2004 and July 2023									
AR1	1.069	0.06	17.678	< 0.001	3693.18	3717.4	− 1839.59	0.152	0.696
AR2	− 0.419	0.066	− 6.359	< 0.001					
AR4	0.15	0.043	3.503	< 0.001					
SAR1	0.143	0.063	2.26	0.024					
Pulse change	− 213.922	419.083	− 0.511	0.61					

### Effect estimations and forecasts using ARFIMA

**Fig. 2:**
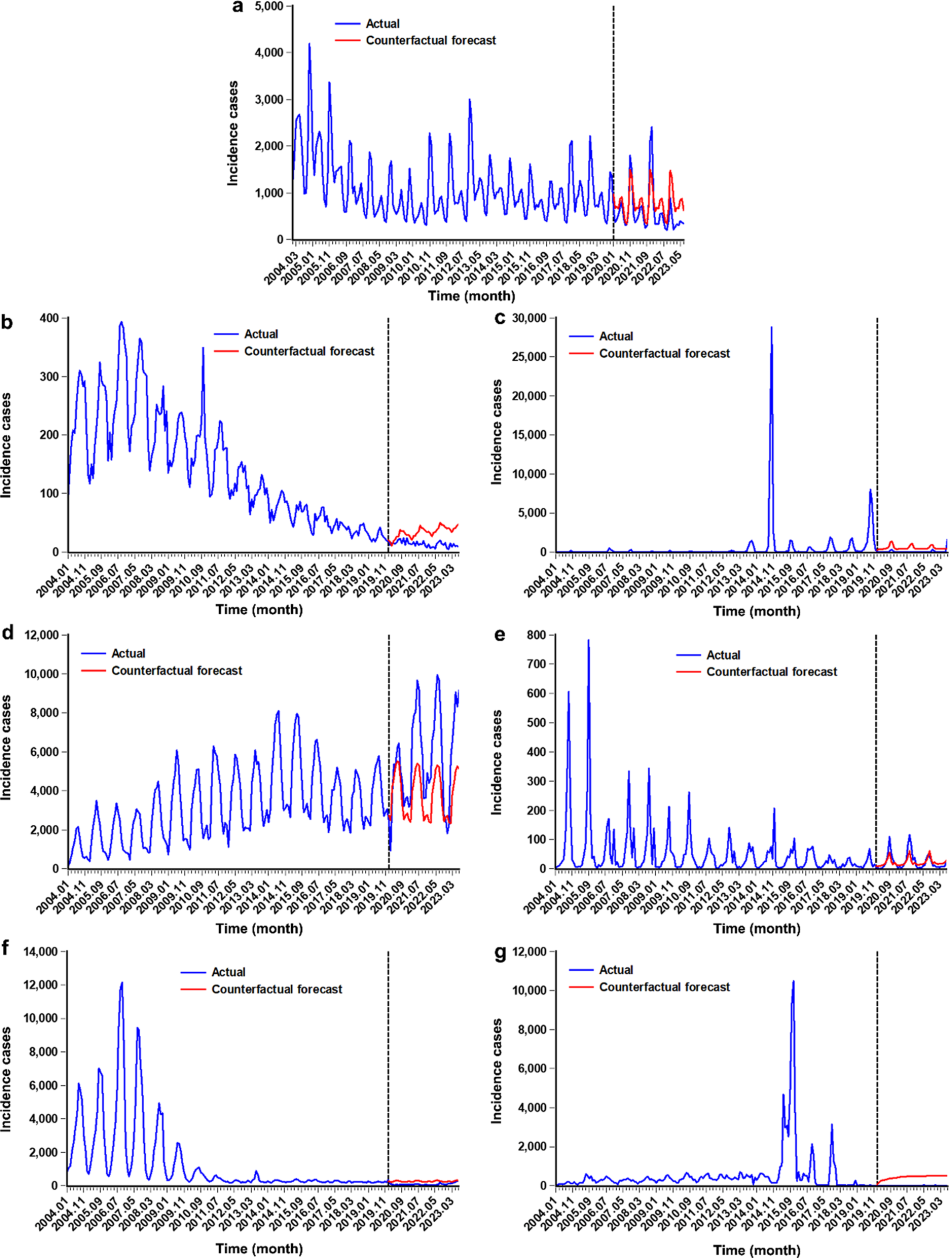
The actual epidemic patterns and counterfactual predictions under the COVID-19-associated public health interventions between January 2020 and July 2023. a Counterfactual prediction for the HFRS incidence series, b counterfactual prediction for the rabies incidence series, c counterfactual prediction for the DF incidence series, d counterfactual prediction for the HB incidence series, e counterfactual prediction for the leptospirosis incidence series, f counterfactual prediction for the malaria incidence series, and g counterfactual prediction for the schistosomiasis incidence series. It can be seen that seemingly the COVID-19-related public health interventions led to a case reduction in HFRS, rabies, DF, malaria, and schistosomiasis incidences except for HB and leptospirosis which showed a rising tendency during the COVID-19 pandemic

**Fig. 3:**
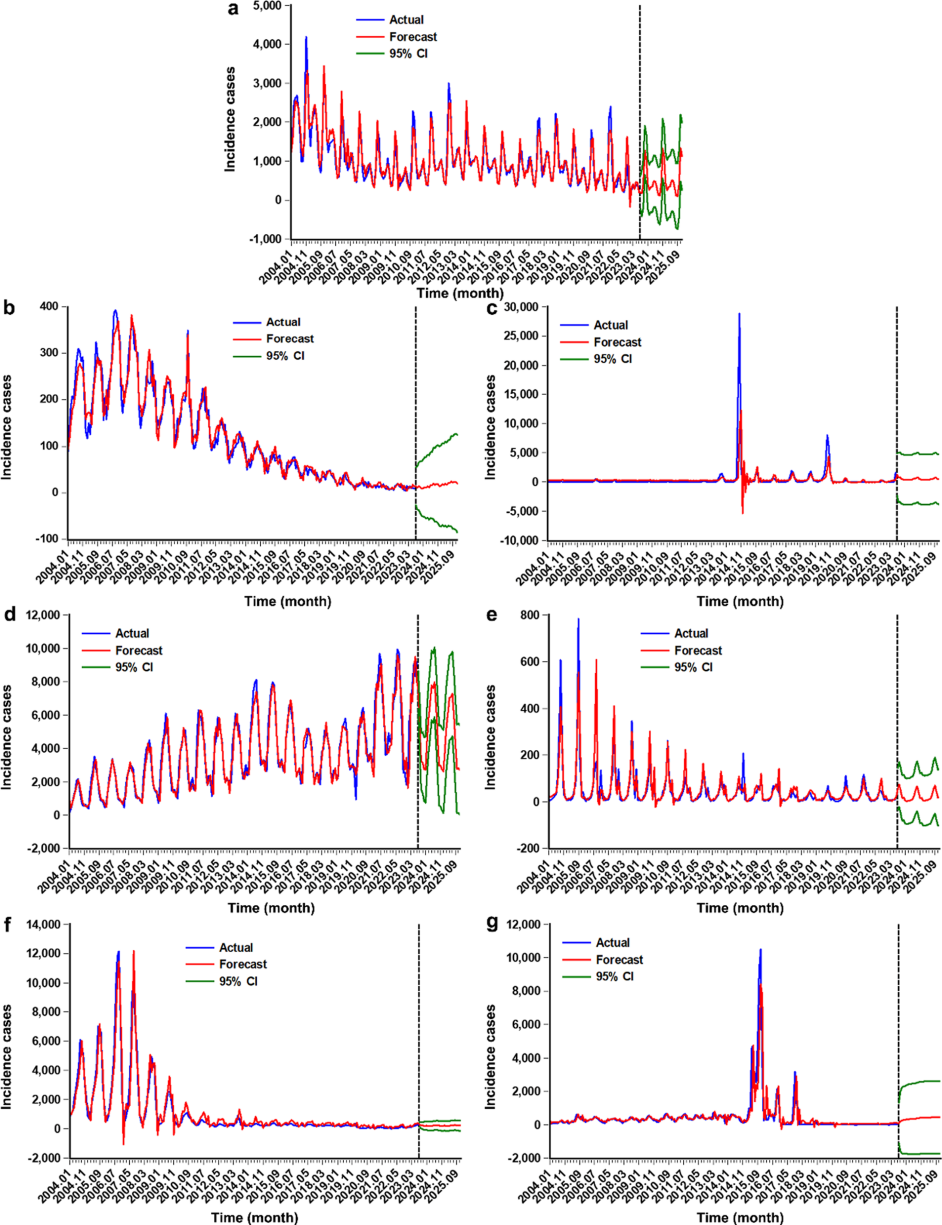
The predicted epidemics from August 2023 to December 2025 under the ARFIMA considering the effects of the COVID-19 outbreak. a Prediction for the HFRS incidence series, b prediction for the rabies incidence series, c prediction for the DF incidence series, d prediction for the HB incidence series, e prediction for the leptospirosis incidence series, f prediction for the malaria incidence series, and g prediction for the schistosomiasis incidence series. As shown, the predicted trends remained at a relative plateau for HFRS, rabies, DF, leptospirosis, malaria, and schistosomiasis except for HB which predicted a decline

To see the effect, we fitted the same ARFIMA model to the different ZVBs incidence series without including the COVID-19-associated public health intervention variable (the pulse pattern created), using only the data up to December 2019. Then, we forecasted the different ZVBs incidence data into 43 months (from January 2020 to July 2023), which allowed us to see how the actual data deviates from what would have been expected without the intervention (i.e. the forecast was deemed as a counterfactual to describe the potential effect of the intervention on the series). By comparing the observed values with the forecasted values (Fig. 2), it appears that the COVID-19-associated public health interventions resulted in a decline in HFRS, rabies, DF, malaria, and schistosomiasis incidences, whereas the HB and leptospirosis incidences exhibited a rising tendency during COVID-19 pandemic. Also, the estimated pulse variable enabled us to quantify the long-term impact of the interventions (Table 2), we found a pulse change of − 83 (95% confidence interval [CI] − 353–189) cases in HFRS, − 3 (95% CI − 33–29) cases in rabies, − 468 (95% CI − 1531–597) cases in DF, 2191 (95% CI 1056–3326) cases in HB, 7 (95% CI − 24–38) cases in leptospirosis, -84 (95% CI − 222–55) cases in malaria, and -214 (95% CI – 1036–608) cases in schistosomiasis. These meant that the COVID-19-related measures led to an average monthly reduction of 83, 3, 468, 84, and 214 cases in HFRS, rabies, DF, malaria, and schistosomiasis, respectively, despite no significance. Further, the predicted figures until December 2025 from the ARFIMA considering the COVID-19 effect were plotted in Fig. 3, suggesting a relative plateau for HFRS, rabies, DF, leptospirosis, malaria, and schistosomiasis besides HB (which predicted a decline) in the next 29 months.

## Discussion

Since the start of 2020, the swift global spread of COVID-19 has led to significant health, social, and economic consequences [2]. Despite some efforts to curb these impacts through the dynamic “zero-COVID” policy, the impact of this on the diseases and their consequences may vary over time. Nevertheless, it has yet to be investigated how COVID-19-related public health interventions have influenced the ZVBs epidemics in the long run. To our knowledge, this is the only study focusing on evaluating the long-term impact of COVID-19-related interventions on ZVBs epidemics and forecasting their epidemics in China using a new ITS of ARFIMA. Our interesting findings were that the public health measures associated with COVID-19 contributed to cutting the ZVBs incidences in some, but not all in the long term. Specifically, these interventions played a lasting negative effect on HFRS, rabies, DF, malaria, and schistosomiasis notwithstanding no statistical significance; however, contrary to such an impact above, there was a rising trend in HB and leptospirosis during the COVID-19 pandemic. A recent study observed that the stringent COVID-19 policy was associated with a remarkable decrease in seven respiratory communicable diseases (including measles, tuberculosis, pertussis, scarlet fever, seasonal influenza, mumps, and rubella) for the year 2020 and 2021 compared to the year 2019 [8]. Also, the WHO reported a continued damaging effect on tuberculosis epidemics [27]. These studies provide additional support for our findings in HFRS, rabies, DF, among others despite the different routes of transmission between respiratory diseases and ZVBs. Besides, a prior study reported that this policy resulted in a sharp decline in these five ZVBs in 2020 [6], also providing additional evidence.

The plausible explanations for the long-term effect may be two-faceted: 1) Positive effects (real reduction). First, the dynamic “zero-COVID” policy has resulted in reduced human movement and outdoor activities [3]. This has led to a decrease in human-host contact, thereby reducing the transmission of these five ZVBs. Second, with increased public health awareness during the pandemic [3, 12], there has been a greater emphasis on vector control measures, contributing to a reduction in vector populations and disease transmission. Third, the promotion of hand hygiene, wearing masks, and maintaining cleanliness has led to a reduced risk of infection for diseases transmitted through contaminated surfaces or vectors [3]. Fourth, most DF and malaria cases were indicated to be imported diseases in China [5, 6], border restrictions and quarantine reduce cross-border movement (a study showed a decrease of 60–80% in international flights during the COVID-19 pandemic [5]), causing the reduction of DF and malaria. (2) Negative effects (under-reporting, late-reporting, or misreporting) [5, 7, 28, 29]. First, the diversion of resources and attention towards COVID-19 response has resulted in the disruption of routine vector surveillance and control programs. This may lead to a decrease in vector monitoring and timely intervention. Second, the focus on COVID-19 care and restrictions on non-emergency healthcare services have limited access to healthcare facilities for individuals with these ZVBs, resulting in delayed diagnosis, treatment, and management of these diseases [28]. Third, the redirection of resources and attention towards COVID-19 surveillance has affected the surveillance systems for these ZVBs, resulting in underreporting or delayed detection of cases [28]. Fourth, persons with ZVBs may be hesitant to undergo medical check-ups because of stringent policy and the requirement for negative nucleic acid testing results [29]. Fifth, the clinical features of these ZVBs, such as fever and cough, bear similarities to those of COVID-19, which raises concerns about stigma [28, 30].

There was a noticeable upward trend in the cases of HB and leptospirosis during the COVID-19 outbreak. Despite limited available evidence regarding the prolonged impact of COVID-19-related interventions on these two diseases, our intriguing findings align closely with studies conducted in Yinchuan [10], Jiangsu [6], Zhejiang [11], and mainland China [7] in 2020. These findings also correspond with reports of a resurgence of leptospirosis during the COVID-19 pandemic in Sri Lanka [28], South India [30], and Tanzania [12]. However, they contrast with the results from Guangdong [5] and the Taiwan region [31], which documented a significant reduction in HB and leptospirosis cases in the year 2020. Geographically, this divergence may be related to the economic status, behavioral lifestyle, eating habits, and climate in these regions [5, 6, 12, 31]. The resurgence of HB during the COVID-19 pandemic may be attributed to a combination of factors, including Lanzhou HB outbreak event (resulting in an upsurge in detection rates and reported cases) [32], increased contact with infected animals (as COVID-19 restrictions were eased or lifted, people had more opportunities for close contact with livestock), healthcare system strain (overwhelming demands on healthcare systems to address COVID-19), diversion of public health efforts (public health resources and efforts were primarily directed towards combating COVID-19), and economic challenges (economic hardships caused by the pandemic may have forced individuals to engage in riskier activities, such as consuming unpasteurized dairy products or handling livestock without adequate protective measures) [5–7, 10, 11]. The rise in leptospirosis cases may be linked to coinfection of leptospirosis and COVID-2019 [30], environmental factors, changes in human behavior and activities [28], sanitation issues [12], healthcare system strain [28], increased awareness [12], and climate-related factors that created conducive conditions for the transmission of the bacteria [12].

Intervention analysis is essential for assessing, improving, and optimizing interventions and policies, facilitating informed decision-making, and promoting transparency and accountability [13, 14]. Selecting the correct analytical method for intervention analysis is a critical step in ensuring effective decision-making and problem-solving [14]. In intervention analysis, it is crucial to factor in trends, seasonality, and autocorrelation [14]. However, a previous review indicated that approximately one-third of intervention analyses did not consider autocorrelation testing, and approximately two-thirds failed to report adjustments for seasonality [33]. Currently, the SRM and ARIMA, as the most popular methods in intervention analysis, have been used to evaluate the effect of large-scale interventions in the public health domain [10, 15–18]. Among them, SRM cannot sufficiently tackle residual autocorrelation and complex seasonal patterns [14], while ARIMA stands as a notable alternative in such scenarios. For example, ARIMA has been widely applied to estimate the impact of COVID-19 on infectious diseases such as hepatitis, tuberculosis, pertussis, malaria, among others [8–10]. Nevertheless, ARIMA is specified for modelling short-term patterns and often generates an over-differencing [19]. Consequently, this study introduced a new long-term ITS analysis method of ARFIMA where the integer differencing of ARIMA is replaced by fractional differencing [20, 21]. By doing so, ARFIMA allows for more flexible modelling of dependencies over longer lags [21], thus addressing the weaknesses of ARIMA. A recent study indicated that ARFIMA could greatly improve the forecasting accuracy in HFRS over the ARIMA [20], which was also corroborated by our study using the data during 2004–2019 (Additional file 1: Table S7). To combat the spread of ZVBs, China has implemented various control and prevention strategies such as vector control measures, surveillance systems, public health education, vaccination programs, and early detection and treatment of cases [34]. We further predicted the seven ZVBs epidemics until December 2025 using the ARFIMA, showing that HB would recede, while the others would remain stable.

There are several limitations. First, the data was obtained from a passive monitoring system, which inevitably leads to under-reporting. Second, the study's design, being ecological, only provides indirect insights. Future research with more rigorous methodologies will be necessary to establish causal relationships conclusively. Third, we were only able to collect monthly and yearly ZVBs cases without specific details such as age, sex, occupation, and vector population associated with ZVBs, which precludes further stratified analysis to identify sensitive individuals and further investigation into the impact of COVID-19-related measures on vector population associated with ZVBs. Fourth, limited data may not adequately capture long-term dependencies, and therefore it is recommended to use a series with at least 100 samples in practical applications. Lastly, this study did not include other factors (e.g., climate and economic conditions [34]) that could potentially impact the spread of ZVBs and the vector population associated with these diseases.

## Conclusions

The interventions implemented to combat COVID-19 in China may have had a lasting preventive effect on HFRS, rabies, DF, malaria, and schistosomiasis beyond the immediate consequences instead of HB and leptospirosis that exhibited an increase during the COVID-19 outbreak. Thrillingly, the HB would be projected to recede, and the others would remain stable in the next 29 months. The ARFIMA holds significant importance and relevance in the field of intervention analysis. Its application allows for a comprehensive understanding of the effects of interventions. By incorporating long memory and fractional differencing, the ARFIMA enables the accurate identification and evaluation of intervention effects, thereby aiding evidence-based policy-making and strategic planning.

### Supplementary Information

**Additional file 1: Table S1. **Decomposed seasonal index for the seven zoonotic and vector-borne diseases based on classical multiplicative decomposition. **Table S2. **Identified possible ARFIMA with the AIC, CAIC, BIC, and LL values. **Table S3.** The resulting ARFIMA modes with their corresponding information criteria for HFRS. **Table S4.** The resulting ARFIMA modes with their corresponding information criteria for rabies. **Table S5.** The resulting ARFIMA modes with their corresponding information criteria for leptospirosis. **Table S6.** The resulting ARFIMA modes with their corresponding information criteria for malaria. **Table S7. **Comparison of the predictive ability for HFRS under the ARIMA and ARFIMA. **Figure S1. **The incidence cases and rates of the seven zoonotic and vector-borne diseases. **Figure S2. **ACF and PACF plots for the seasonally and non-seasonally differenced HFRS incidence series. The significant spikes at lags 2 and 3 in the PACF indicate that the maximum orders may be 3 in the non-seasonal AR component, and the significant spike at lag 12 in the ACF suggests that the maximum orders may be 1 in the seasonal AR component. The significant spikes at lags 2 and 3 in the ACF suggest that the maximum orders may be 3 in the non-seasonal MA component, and the significant spike at lag 12 in the ACF suggests that the maximum orders may be 1 in the seasonal MA component. **Figure S3. **ACF and PACF analyses for the errors from the ARFIMA. a HFRS series residuals, b Rabies residual series, c DF residual series, d HB residual series, e Leptospirosis residual series, f Malaria residual series, and g Schistosomiasis residual series. Here the correlogram showed that few spikes exceeded the estimated significance limits, which is also reasonable in that some high-order correlations easily exceed that by chance alone, suggesting that there is little evidence of non-white noise in the residual series of the seven zoonotic and vector-borne diseases.

## Data Availability

All the data supporting the findings of the work are contained within the study.

## Abbreviations

ZVBs: Zoonotic and vector-borne infectious diseases
ARFIMA: Autoregressive fractionally integrated moving average
ARIMA: Autoregressive integrated moving average
SRM: Segmented regression model
ITS: Interrupted time series
AR: Autoregressive model
MA: Moving average model
HFRS: Hemorrhagic fever with renal syndrome
DF: Dengue fever
HB: Human brucellosis
CI: Confidence interval
AIC: Akaike's Information Criterion
BIC: Bayesian information criterion
ACF: Autocorrelation function
PACF: Partial autocorrelation function
H: Hurst exponent
HP: Hodrick-Prescott
SI: Seasonal index
LL: Log-likelihood
