# Supplementary material for: Evaluating the long-term impact of COVID-19-associated public health interventions on zoonotic and vector-borne diseases in China: an interrupted time series analysis
Source: J Transl Med. 2024 Jan 20;22:81. doi: 10.1186/s12967-024-04855-y (PMC10799468; doi:10.1186/s12967-024-04855-y)
Supplement: Supplementary file 1 — Additional file 1: Table S1. Decomposed seasonal index for the seven zoonotic and vector-borne diseases based on classical multiplicative decomposition. Table S2. Identified possible ARFIMA with the AIC, CAIC, BIC, and LL values. Table S3. The resulting ARFIMA modes with their corresponding information criteria for HFRS. Table S4. The resulting ARFIMA modes with their corresponding information criteria for rabies. Table S5. The resulting ARFIMA modes with their corresponding information criteria for leptospirosis. Table S6. The resulting ARFIMA modes with their corresponding information criteria for malaria. Table S7. Comparison of the predictive ability for HFRS under the ARIMA and ARFIMA. Figure S1. The incidence cases and rates of the seven zoonotic and vector-borne diseases. Figure S2. ACF and PACF plots for the seasonally and non-seasonally differenced HFRS incidence series. The significant spikes at lags 2 and 3 in the PACF indicate that the maximum orders may be 3 in the non-seasonal AR component, and the significant spike at lag 12 in the ACF suggests that the maximum orders may be 1 in the seasonal AR component. The significant spikes at lags 2 and 3 in the ACF suggest that the maximum orders may be 3 in the non-seasonal MA component, and the significant spike at lag 12 in the ACF suggests that the maximum orders may be 1 in the seasonal MA component. Figure S3. ACF and PACF analyses for the errors from the ARFIMA. a HFRS series residuals, b Rabies residual series, c DF residual series, d HB residual series, e Leptospirosis residual series, f Malaria residual series, and g Schistosomiasis residual series. Here the correlogram showed that few spikes exceeded the estimated significance limits, which is also reasonable in that some high-order correlations easily exceed that by chance alone, suggesting that there is little evidence of non-white noise in the residual series of the seven zoonotic and vector-borne diseases. [file 12967_2024_4855_MOESM1_ESM.docx]

**Supplementary material: Evaluating the long-term impact of** **COVID-19-associated public health interventions on zoonotic and vector-borne diseases in China: an interrupted time series analysis**

**Table S1** Decomposed seasonal index for the seven zoonotic and vector-borne diseases based on classical multiplicative decomposition

| Month | HFRS | Rabies | DF | HB | Leptospirosis | Malaria | Schistosomiasis |
| --- | --- | --- | --- | --- | --- | --- | --- |
| January | 1.005 | 0.831 | 0.096 | 0.492 | 0.300 | 0.760 | 0.491 |
| February | 0.687 | 0.695 | 0.061 | 0.533 | 0.121 | 0.625 | 0.465 |
| March | 0.780 | 0.703 | 0.093 | 1.067 | 0.152 | 0.589 | 0.815 |
| April | 0.823 | 0.750 | 0.099 | 1.300 | 0.161 | 0.747 | 0.864 |
| May | 1.017 | 0.864 | 0.190 | 1.514 | 0.283 | 1.051 | 0.989 |
| June | 1.110 | 1.045 | 0.236 | 1.627 | 0.552 | 1.282 | 0.971 |
| July | 0.798 | 1.131 | 0.416 | 1.563 | 0.987 | 1.412 | 1.179 |
| August | 0.486 | 1.285 | 1.093 | 1.279 | 1.984 | 1.477 | 1.299 |
| September | 0.454 | 1.257 | 3.512 | 0.853 | 3.449 | 1.272 | 1.263 |
| October | 0.930 | 1.264 | 4.294 | 0.559 | 1.785 | 1.157 | 1.399 |
| November | 2.096 | 1.115 | 1.615 | 0.601 | 0.877 | 0.882 | 1.094 |
| December | 1.815 | 1.059 | 0.293 | 0.614 | 1.349 | 0.745 | 1.170 |

**Table S****2** Identified possible ARFIMA with the AIC, CAIC, BIC, and LL values

| Models | AIC | CAIC | BIC | LL |
| --- | --- | --- | --- | --- |
|  |  |  |  |  |
| ARFIMA(2,d^*^,1)(1,D*,1) | 3037 | 3035.53 | 3060.82 | -1511.5 |
| ARFIMA(3, d^*^,1)(3,D*,0)_12_ | 2007.61 | 2008.51 | 2032.60 | -995.80 |
| ARFIMA(3,d^*^,1)(2,D* ,0)_12_ | 2018.84 | 2019.54 | 2040.71 | -1002.71 |
| ARFIMA(3,d^*^,1)(1,D*,0)_12_ | 2050.74 | 2051.26 | 2069.49 | -1019.37 |
| ARFIMA(3,d^*^,1)(1,D*,1)_12_ | 2014.59 | 2015.29 | 2036.46 | -1000.30 |
| ARFIMA(2,d^*^,1)(3,D*,0)_12_ | 2014.53 | 2015.23 | 2036.40 | -1000.27 |
| ARFIMA(1,d^*^,1)(3,D*,0)_12_ | 2014.55 | 2015.07 | 2033.29 | -1001.27 |

**Table S3** The resulting ARFIMA modes with their corresponding information criteria for HFRS

| Modes | AIC | BIC | LL |
| --- | --- | --- | --- |
| Mode 1 | 2574.86 | 2609.45 | -1277.4 |
| Mode 2 | 2574.86 | 2609.46 | -1277.4 |
| Mode 3 | 2575.13 | 2609.72 | -1277.6 |
| Mode 4 | 2575.18 | 2609.78 | -1277.6 |
| Mode 5 | 2575.18 | 2609.78 | -1277.6 |
| Mode 6 | 2575.19 | 2609.79 | -1277.6 |
| Mode 7 | 2575.22 | 2609.81 | -1277.6 |
| Mode 8 | 2575.23 | 2609.83 | -1277.6 |
| Mode 9 | 2575.28 | 2609.87 | -1277.6 |
| Mode 10 | 2575.29 | 2609.88 | -1277.6 |
| Mode 11 | 2575.3 | 2609.89 | -1277.7 |
| Mode 12 | 2575.3 | 2609.9 | -1277.7 |
| Mode 13 | 2575.3 | 2609.9 | -1277.7 |
| Mode 14 | 2575.3 | 2609.9 | -1277.7 |
| Mode 15 | 2575.33 | 2609.92 | -1277.7 |
| Mode 16 | 2575.33 | 2609.93 | -1277.7 |
| Mode 17 | 2575.33 | 2609.93 | -1277.7 |
| Mode 18 | 2575.34 | 2609.93 | -1277.7 |
| Mode 19 | 2575.38 | 2609.98 | -1277.7 |
| Mode 20 | 2575.41 | 2610 | -1277.7 |
| Mode 21 | 2575.43 | 2610.03 | -1277.7 |
| Mode 22 | 2575.47 | 2610.06 | -1277.7 |
| Mode 23 | 2575.48 | 2610.08 | -1277.7 |
| Mode 24 | 2575.57 | 2610.17 | -1277.8 |
| Mode 25 | 2575.6 | 2610.2 | -1277.8 |
| Mode 26 | 2576.01 | 2610.61 | -1278 |
| Mode 27 | 2576.57 | 2611.16 | -1278.3 |
| Mode 28 | 2576.61 | 2611.21 | -1278.3 |
| Mode 29 | 2577.26 | 2611.85 | -1278.6 |
| Mode 30 | 2577.36 | 2611.96 | -1278.7 |
| Mode 31 | 2577.74 | 2612.34 | -1278.9 |
| Mode 32 | 2578.14 | 2612.74 | -1279.1 |
| Mode 33 | 2578.48 | 2613.08 | -1279.2 |
| Mode 34 | 2578.58 | 2613.18 | -1279.3 |
| Mode 35 | 2578.67 | 2613.27 | -1279.3 |
| Mode 36 | 2578.9 | 2613.49 | -1279.5 |
| Mode 37 | 2578.98 | 2613.57 | -1279.5 |
| Mode 38 | 2579.32 | 2613.91 | -1279.7 |
| Mode 39 | 2579.33 | 2613.93 | -1279.7 |
| Mode 40 | 2579.37 | 2613.96 | -1279.7 |
| Mode 41 | 2579.37 | 2613.97 | -1279.7 |
| Mode 42 | 2579.42 | 2614.02 | -1279.7 |
| Mode 43 | 2579.46 | 2614.06 | -1279.7 |
| Mode 44 | 2579.47 | 2614.07 | -1279.7 |
| Mode 45 | 2579.53 | 2614.13 | -1279.8 |
| Mode 46 | 2579.72 | 2614.32 | -1279.9 |
| Mode 47 | 2579.88 | 2614.47 | -1279.9 |
| Mode 48 | 2579.95 | 2614.54 | -1280 |
| Mode 49 | 2580 | 2614.59 | -1280 |
| Mode 50 | 2580 | 2614.6 | -1280 |
| Mode 51 | 2580.02 | 2614.61 | -1280 |
| Mode 52 | 2580.06 | 2614.65 | -1280 |
| Mode 53 | 2580.09 | 2614.68 | -1280 |
| Mode 54 | 2580.13 | 2614.73 | -1280.1 |
| Mode 55 | 2580.14 | 2614.73 | -1280.1 |
| Mode 56 | 2580.14 | 2614.74 | -1280.1 |
| Mode 57 | 2580.17 | 2614.76 | -1280.1 |
| Mode 58 | 2580.27 | 2614.87 | -1280.1 |
| Mode 59 | 2580.27 | 2614.87 | -1280.1 |
| Mode 60 | 2580.3 | 2614.89 | -1280.2 |
| Mode 61 | 2580.31 | 2614.91 | -1280.2 |
| Mode 62 | 2580.32 | 2614.91 | -1280.2 |
| Mode 63 | 2580.33 | 2614.92 | -1280.2 |
| Mode 64 | 2580.37 | 2614.97 | -1280.2 |
| Mode 65 | 2580.54 | 2615.13 | -1280.3 |
| Mode 66 | 2580.59 | 2615.19 | -1280.3 |
| Mode 67 | 2580.65 | 2615.24 | -1280.3 |
| Mode 68 | 2580.71 | 2615.3 | -1280.4 |
| Mode 69 | 2580.73 | 2615.32 | -1280.4 |
| Mode 70 | 2580.76 | 2615.35 | -1280.4 |
| Mode 71 | 2580.97 | 2615.56 | -1280.5 |
| Mode 72 | 2581.13 | 2615.73 | -1280.6 |
| Mode 73 | 2581.17 | 2615.77 | -1280.6 |
| Mode 74 | 2581.67 | 2616.27 | -1280.8 |
| Mode 75 | 2582.37 | 2616.97 | -1281.2 |
| Mode 76 | 2582.49 | 2617.09 | -1281.3 |
| Mode 77 | 2582.83 | 2617.42 | -1281.4 |
| Mode 78 | 2582.88 | 2617.48 | -1281.4 |
| Mode 79 | 2582.92 | 2617.51 | -1281.5 |
| Mode 80 | 2582.93 | 2617.52 | -1281.5 |
| Mode 81 | 2582.93 | 2617.53 | -1281.5 |
| Mode 82 | 2582.93 | 2617.53 | -1281.5 |
| Mode 83 | 2582.94 | 2617.54 | -1281.5 |
| Mode 84 | 2582.94 | 2617.54 | -1281.5 |
| Mode 85 | 2582.96 | 2617.56 | -1281.5 |
| Mode 86 | 2582.97 | 2617.56 | -1281.5 |
| Mode 87 | 2583.05 | 2617.65 | -1281.5 |
| Mode 88 | 2583.06 | 2617.66 | -1281.5 |
| Mode 89 | 2583.06 | 2617.66 | -1281.5 |
| Mode 90 | 2583.1 | 2617.7 | -1281.6 |
| Mode 91 | 2583.14 | 2617.73 | -1281.6 |
| Mode 92 | 2583.34 | 2617.94 | -1281.7 |
| Mode 93 | 2583.41 | 2618.01 | -1281.7 |
| Mode 94 | 2583.6 | 2618.19 | -1281.8 |
| Mode 95 | 2583.94 | 2618.54 | -1282 |
| Mode 96 | 2583.95 | 2618.54 | -1282 |
| Mode 97 | 2584.02 | 2618.62 | -1282 |
| Mode 98 | 2584.04 | 2618.63 | -1282 |
| Mode 99 | 2585.22 | 2619.82 | -1282.6 |
| Mode 100 | 2585.75 | 2620.35 | -1282.9 |
| Mode 101 | 2585.77 | 2620.36 | -1282.9 |
| Mode 102 | 2585.77 | 2620.37 | -1282.9 |
| Mode 103 | 2585.78 | 2620.37 | -1282.9 |
| Mode 104 | 2585.78 | 2620.38 | -1282.9 |
| Mode 105 | 2585.78 | 2620.38 | -1282.9 |
| Mode 106 | 2585.79 | 2620.38 | -1282.9 |
| Mode 107 | 2586.07 | 2620.67 | -1283 |
| Mode 108 | 2588.32 | 2622.91 | -1284.2 |
| Mode 109 | 2591.92 | 2626.52 | -1286 |
| Mode 110 | 2593.12 | 2627.71 | -1286.6 |
| Mode 111 | 2593.79 | 2628.38 | -1286.9 |
| Mode 112 | 2595.98 | 2630.57 | -1288 |
| Mode 113 | 2596.58 | 2631.18 | -1288.3 |
| Mode 114 | 2599.2 | 2633.8 | -1289.6 |
| Mode 115 | 2599.28 | 2633.88 | -1289.6 |
| Mode 116 | 2599.69 | 2634.29 | -1289.8 |
| Mode 117 | 2602.93 | 2637.53 | -1291.5 |
| Mode 118 | 2622.91 | 2657.51 | -1301.5 |
| Mode 119 | 2699.11 | 2733.71 | -1339.6 |
| Mode 120 | 2732.95 | 2767.54 | -1356.5 |
| Mode 121 | 2780.06 | 2814.66 | -1380 |
| Mode 122 | 2804.46 | 2839.05 | -1392.2 |

**Table S4** The resulting ARFIMA modes with their corresponding information criteria for rabies

| Modes | AIC | BIC | LL |
| --- | --- | --- | --- |
| Mode 1 | 1518.222 | 1552.818 | -749.111 |
| Mode 2 | 1518.226 | 1552.822 | -749.113 |
| Mode 3 | 1518.227 | 1552.823 | -749.114 |
| Mode 4 | 1518.233 | 1552.828 | -749.116 |
| Mode 5 | 1518.234 | 1552.83 | -749.117 |
| Mode 6 | 1518.243 | 1552.838 | -749.121 |
| Mode 7 | 1518.246 | 1552.842 | -749.123 |
| Mode 8 | 1518.253 | 1552.849 | -749.127 |
| Mode 9 | 1518.264 | 1552.859 | -749.132 |
| Mode 10 | 1518.264 | 1552.86 | -749.132 |
| Mode 11 | 1518.285 | 1552.881 | -749.142 |
| Mode 12 | 1518.286 | 1552.882 | -749.143 |
| Mode 13 | 1518.3 | 1552.896 | -749.15 |
| Mode 14 | 1518.306 | 1552.902 | -749.153 |
| Mode 15 | 1518.317 | 1552.913 | -749.158 |
| Mode 16 | 1518.319 | 1552.915 | -749.159 |
| Mode 17 | 1518.327 | 1552.923 | -749.164 |
| Mode 18 | 1518.329 | 1552.925 | -749.164 |
| Mode 19 | 1518.336 | 1552.932 | -749.168 |
| Mode 20 | 1518.337 | 1552.933 | -749.168 |
| Mode 21 | 1518.34 | 1552.936 | -749.17 |
| Mode 22 | 1518.349 | 1552.945 | -749.174 |
| Mode 23 | 1518.349 | 1552.945 | -749.175 |
| Mode 24 | 1518.35 | 1552.946 | -749.175 |
| Mode 25 | 1518.354 | 1552.95 | -749.177 |
| Mode 26 | 1518.355 | 1552.951 | -749.177 |
| Mode 27 | 1518.359 | 1552.955 | -749.18 |
| Mode 28 | 1591.328 | 1625.924 | -785.664 |
| Mode 29 | 1615.087 | 1649.683 | -797.544 |
| Mode 30 | 1620.322 | 1654.918 | -800.161 |
| Mode 31 | 1820.194 | 1854.789 | -900.097 |

**Table S5** The resulting ARFIMA modes with their corresponding information criteria for leptospirosis

| Modes | AIC | BIC | LL |
| --- | --- | --- | --- |
| Mode 1 | 1830.26 | 1864.86 | -905.13 |
| Mode 2 | 1830.26 | 1864.86 | -905.13 |
| Mode 3 | 1830.27 | 1864.86 | -905.13 |
| Mode 4 | 1830.27 | 1864.87 | -905.13 |
| Mode 5 | 1830.27 | 1864.87 | -905.14 |
| Mode 6 | 1830.28 | 1864.87 | -905.14 |
| Mode 7 | 1830.28 | 1864.87 | -905.14 |
| Mode 8 | 1830.28 | 1864.87 | -905.14 |
| Mode 9 | 1830.28 | 1864.88 | -905.14 |
| Mode 10 | 1830.28 | 1864.88 | -905.14 |
| Mode 11 | 1852.82 | 1887.41 | -916.41 |
| Mode 12 | 1855.14 | 1889.74 | -917.57 |
| Mode 13 | 1864.11 | 1898.71 | -922.06 |

**Table S6** The resulting ARFIMA modes with their corresponding information criteria for malaria

| Modes | AIC | BIC | LL |
| --- | --- | --- | --- |
| Mode 1 | 1430.06 | 1457.91 | -706.03 |
| Mode 2 | 1430.09 | 1457.93 | -706.04 |
| Mode 3 | 1430.44 | 1458.28 | -706.22 |
| Mode 4 | 1430.89 | 1458.74 | -706.45 |
| Mode 5 | 1430.93 | 1458.78 | -706.47 |
| Mode 6 | 1431.47 | 1459.32 | -706.74 |
| Mode 7 | 1432.74 | 1460.59 | -707.37 |
| Mode 8 | 1433.05 | 1460.89 | -707.52 |
| Mode 9 | 1433.24 | 1461.08 | -707.62 |
| Mode 10 | 1435.61 | 1463.45 | -708.81 |
| Mode 11 | 1436.15 | 1463.99 | -709.07 |
| Mode 12 | 1436.82 | 1464.67 | -709.41 |
| Mode 13 | 1437.29 | 1465.13 | -709.64 |
| Mode 14 | 1438.69 | 1466.53 | -710.35 |
| Mode 15 | 1439.38 | 1467.23 | -710.69 |
| Mode 16 | 1441.62 | 1469.47 | -711.81 |
| Mode 17 | 1442.11 | 1469.95 | -712.06 |
| Mode 18 | 1443.47 | 1471.31 | -712.73 |

**Table S7** Comparison of the predictive ability for HFRS under the ARIMA and ARFIMA

| Models | MAD | MAPE | RMSE | RMSPE | MER |
| --- | --- | --- | --- | --- | --- |
| ARIMA(0,1,3)(1,1,0)_12_ | 403.329 | 0.432 | 464.414 | 0.466 | 0.016 |
| ARFIMA(0,0.324,3)(1,-0.245,0)_12_ | 212.892 | 0.183 | 309.826 | 0.215 | 0.007 |

MAD mean absolute deviation, MAPE mean absolute percentage error, RMSE root mean square error, RMSPE root mean square percentage error, MER mean error rate.


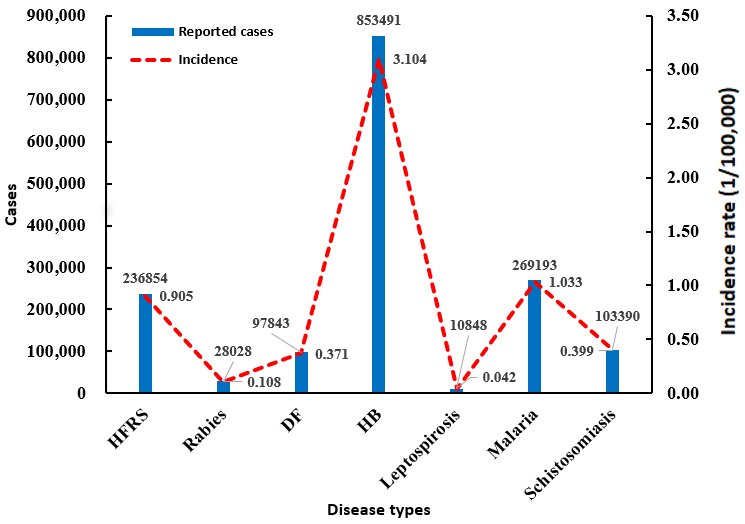


**Figure S1** The incidence cases and rates of the seven zoonotic and vector-borne diseases


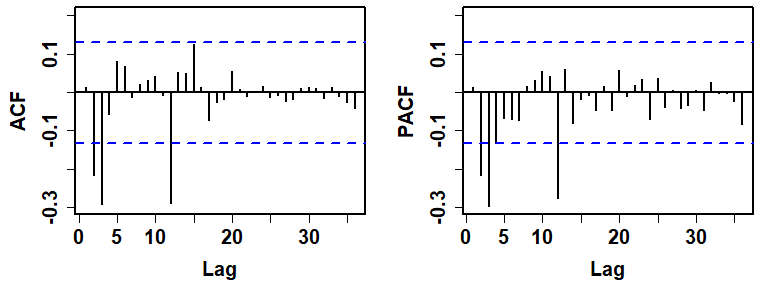


**Figure S2** ACF and PACF plots for the seasonally and non-seasonally differenced HFRS incidence series. The significant spikes at lags 2 and 3 in the PACF indicate that the maximum orders may be 3 in the non-seasonal AR component, and the significant spike at lag 12 in the ACF suggests that the maximum orders may be 1 in the seasonal AR component. The significant spikes at lags 2 and 3 in the ACF suggest that the maximum orders may be 3 in the non-seasonal MA component, and the significant spike at lag 12 in the ACF suggests that the maximum orders may be 1 in the seasonal MA component.


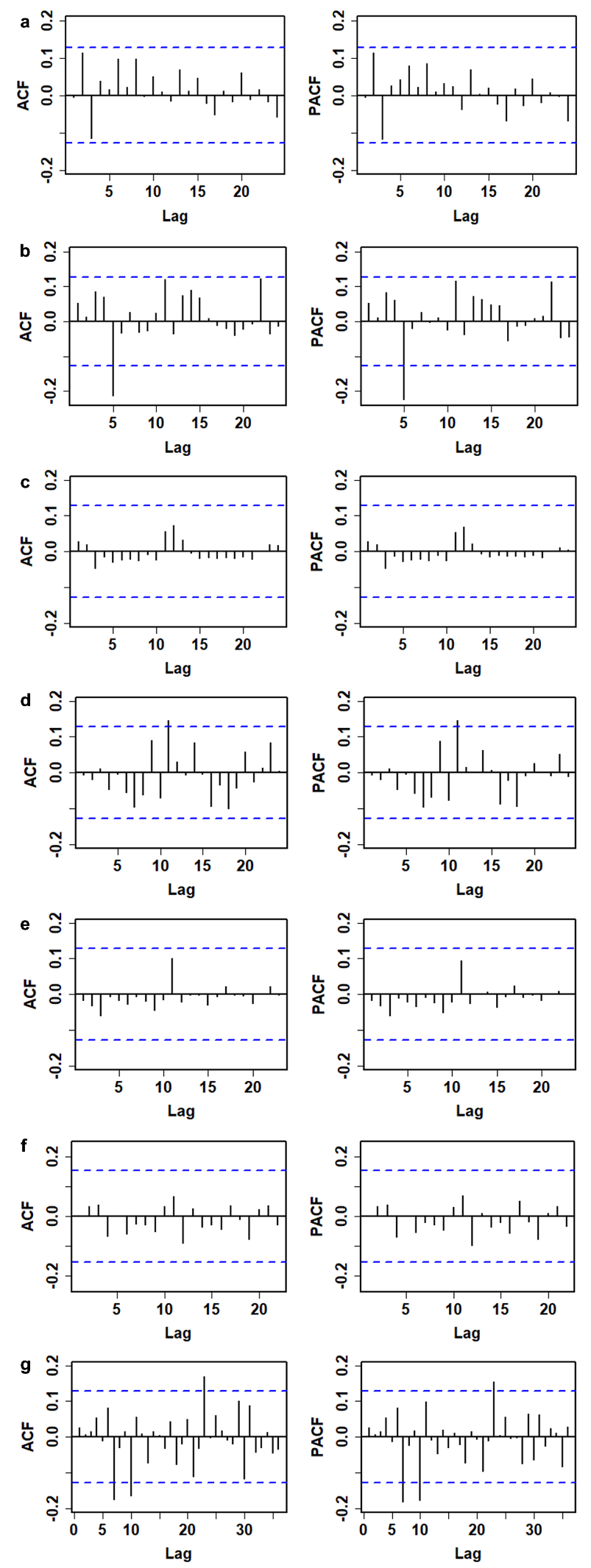


**Figure S3** ACF and PACF analyses for the errors from the ARFIMA. a HFRS series residuals, b Rabies residual series, c DF residual series, d HB residual series, e Leptospirosis residual series, f Malaria residual series, and g Schistosomiasis residual series. Here the correlogram showed that few spikes exceeded the estimated significance limits, which is also reasonable in that some high-order correlations easily exceed that by chance alone, suggesting that there is little evidence of non-white noise in the residual series of the seven zoonotic and vector-borne diseases.
